# Clinical Results and Complications of Lower Limb Lengthening for Fibular Hemimelia: A Report of Eight Cases

**DOI:** 10.1097/MD.0000000000003787

**Published:** 2016-05-27

**Authors:** Kenichi Mishima, Hiroshi Kitoh, Koji Iwata, Masaki Matsushita, Yoshihiro Nishida, Tadashi Hattori, Naoki Ishiguro

**Affiliations:** From the Department of Orthopaedic Surgery (KM, HK, MM, YN, NI), Nagoya University Graduate School of Medicine, Nagoya; and Department of Orthopaedic Surgery (KI, TH), Aichi Children's Health and Medical Center, Obu, Aichi, Japan.

## Abstract

Fibular hemimelia is a rare but the most common congenital long bone deficiency, encompassing a broad range of anomalies from isolated fibular hypoplasia up to substantial femoral and tibial shortening with ankle deformity and foot deficiency. Most cases of fibular hemimelia manifest clinically significant leg length discrepancy (LLD) with time that requires adequate correction by bone lengthening for stable walking. Bone lengthening procedures, especially those for pathological bones, are sometimes associated with severe complications, such as delayed consolidation, fractures, and deformities of the lengthened bones, leading to prolonged healing time and residual LLD at skeletal maturity. The purpose of this study was to review our clinical results of lower limb lengthening for fibular hemimelia.

This study included 8 Japanese patients who diagnosed with fibular hemimelia from physical and radiological findings characteristic of fibular hemimelia and underwent single or staged femoral and/or tibial lengthening during growth or after skeletal maturity. LLD, state of the lengthened callus, and bone alignment were evaluated with full-length radiographs of the lower limb. Previous interventions, associated congenital anomalies, regenerate fractures were recorded with reference to medical charts and confirmed on appropriate radiographs. Successful lengthening was defined as the healing index <50 days/cm without regenerate fractures.

A significant difference was observed in age at surgery between successful and unsuccessful lengthening. The incidence of regenerate fractures was significantly correlated with callus maturity before frame removal. LLD was corrected within 11 mm, whereas mechanical axis deviated laterally.

Particular attention should be paid to the status of callus maturation and the mechanical axis deviation during the treatment period in fibular hemimelia.

## INTRODUCTION

Fibular hemimelia represents a spectrum of congenital disorders characterized by various severity of fibular hypoplasia with or without associated anatomical abnormalities in the lower extremity. It often involves femoral and tibial hypoplasia, causing clinically significant leg length discrepancy (LLD), hypoplasia of the lateral femoral condyle, and anteromedial bowing of the tibia, leading to genu valgum or knock-knee, valgus, or varus deformity of the ankle and deficiency of the lateral ray of the foot, precluding plantigrade walking, and ball-and-socket type of the ankle and tarsal coalition of the foot, possibly generating unpleasant pains on walking.^1^ Fibular hemimelia is one of the most common congenital deficiencies of the long bones with an estimated incidence between 7.4 and 20 per 1 million live births.^2–5^ Almost all cases are sporadic, and the etiology remains elusive. Most cases of fibular hemimelia cause clinically significant LLD due to the associated hypoplasia of the ipsilateral tibia and/or femur, and require corrective surgical procedures such as bone lengthening for the affected limb and/or epiphysiodesis for the healthy side.^6^ In general, LLD greater than 2 cm can deteriorate trunk balance and load asymmetrical stress to lower limb joints and pelvic girdle, increasing the likelihood of early degenerative changes.^7^ Bone-lengthening procedure can bring about major complications that often require surgical interventions to resolve, including delayed consolidation, nonunion, refracture, joint stiffness, hardware failure, late bowing, and neurovascular injury. Epiphysiodesis is another surgical procedure for correcting LLD that slows or halts the growing of the longer side of the lower extremity. Longitudinal bone growth is inhibited by fixing the epiphyseal growth plate with use of screws, staples, and plates. Bone lengthening for fibular hemimelia has been reported to be associated with higher incidence of the major complications, including delayed consolidation (healing index (HI) ≥50 days/cm), regenerating bone fractures, and valgus malalignment of the affected limb.^8–11^ HI is defined as the number of days of treatment period with an external fixator divided by the length gained in centimeters (days/cm). Significant LLD after bone lengthening occasionally remains in fibular hemimelia because underlying poor potential of bone regeneration makes targeted amount of lengthening difficult.

We reviewed our clinical results of lower limb lengthening for fibular hemimelia, highlighting the incidence and characteristics of the major complications, which would contribute to successful treatment of limb lengthening for this intractable disorder.

## MATERIALS AND METHODS

### Patient Cohort

This study is a retrospective case series study. With the institutional review board approval, we retrospectively reviewed clinical records and radiographs of patients with fibular hemimelia who were treated in a tertiary hospital and a pediatric hospital between 1991 and 2012. A total of 8 consecutive patients with fibular hemimelia were included in this study. Inclusion criteria were as follows: patients diagnosed with fibular hemimelia on the basis of various degrees of fibular hypoplasia and the concomitant congenital abnormalities; patients undergoing lower limb lengthening for 2 cm or more of LLD. Patients having inadequate clinical records or lacked serial teleoroentgenograms of the lower limb were excluded from the study. All patients were otherwise normal with no remarkable family history. The concomitant abnormalities in the knee (hypoplasia of the lateral femoral condyle), lower leg (anteromedial bowing of the tibia), ankle (ball and socket joint), and foot (deficiency of the lateral ray and tarsal coalition) were also confirmed radiologically.^1^ Clinical information and treatment for associated foot deformities were obtained from the medical records. Patients were classified according to the Achterman–Kalamchi classification. The classification system categorizes patients into 3 types: Type I-A, the proximal fibular epiphysis is located distal to the proximal tibial physis, whereas the distal fibular physis proximal to the dome of the talus; Type I-B, there is partial deficiency of the fibula, with its proximal portion exhibiting 30% to 50% of absence of whole fibula and its distal portion not supporting the ankle; Type II, there is complete absence of the fibula or only a distal vestigial fragment.^12^ No patients underwent concomitant or staged epiphysiodesis of the healthy side during follow-up periods.

### Bone-Lengthening Procedure

All but 1 bone-lengthening procedures were performed by means of monolateral fixator devices, whereas 1 tibial lengthening was done with Taylor Spatial Frame (Smith & Nephew, Memphis, TN), which we introduced since 2012 for the correction of complicated long bone deformity associated with or without clinically significant LLD. A monolateral external fixator (Dynafix Rail System, Electro-Biology Inc [EBI]/Zimmer Biomet; Warsaw, Indiana, USA) was attached to a patient's lower leg parallel to the mechanical axis of the femur and the anatomical axis of the tibia using an image intensifier. Minimally invasive osteotomy with multiple drilling and chiseling was performed in the vicinity of the proximal metaphysis to minimize damage to the adjacent periosteum. During bone lengthening of the tibia, a 2-cm-long partial excision of the mid-fibula was conducted in all cases of type Ia and in some of type Ib at the surgeon's discretion, but in none of type II. Gradual distraction by 0.5 mm twice a day was commenced after confirmation of the appearance of cloudy callus at the osteotomy site on radiographs. We slowed or halted distraction when the lengthened callus was getting thin and sparse on X-rays. Once a targeted amount of lengthening was achieved, the frame was firmly secured for a few months so that the regenerating bone could consolidate. The flame was subsequently loosened for dynamization to allow compression force across the regenerate. Full-weight bearing was allowed throughout the treatment period. All patients were subjected to the low-intensity pulsed ultrasound (LIPUS) therapy using Sonic Accelerated Fracture Healing System (SAFHS; Teijin Pharma Limited, Osaka, Japan) during the neutralizing and dynamization periods. We have routinely utilized it to facilitate callus maturation and to shorten healing time in pathological bone-lengthening procedures. When the regenerate bone showed continuous shape and similar density to the adjacent cortical bone on radiographs, we removed the fixator with the bone screws left over. For the patients who had the regenerating bone fractures during the observation period, we reattached the fixator to the remaining screws. Otherwise, we removed the screws following 1 to 2 weeks of the further follow-up. Instead of casting, the routine use of a functional brace was commenced immediately after the screw removal.

Bone lengthening of the femur and tibia was scheduled when LLD reached nearly 5 cm or more during the first decade of life. The choice of concomitant or staged lengthening depended on patients’ preferences. In the teenage and adult patients, the amount of lengthening was planned to be equal to LLD. In the younger patients, over-lengthening was planned to resolve the predicted LLD at skeletal maturity, which was roughly estimated with the use of consistent longitudinal data of LLD on the assumption of constant increased rate until puberty.^12^

### Radiological Evaluation

LLD was evaluated on a picture archiving and communication system (PACS) with the use of an anteroposterior full-length lying radiograph of both lower extremities described previously.^13^ The lengths of the femur and tibia were defined as the distances from the superior boarder of the femoral head to the distal end of the medial femoral condyle and the intercondylar eminence of the tibia to the midpoint of the ankle mortise, respectively. LLD was then determined by sum of the differences of the femoral and tibial lengths on each side.

Lengthening ratio was obtained by dividing the amount of lengthening by the original length of each bone. Hypoplasia of the lateral femoral condyle was defined as 0.80 or less of the condylar height ratio using the technique described by Boakes et al.^2^ In brief, we measured the greatest perpendicular length from the physis to the subchondral line at both the lateral and the medial condyle. The ratio was determined by dividing the lateral height by the medial height. With regard to tarsal coalition, we examined lateral radiographs of the ankle to find the C sign that represents subtalar coalition as described previously.^14^ Gross deficiency of the lateral ray of the foot was radiologically confirmed using dorsoplantar radiograph of the foot. Mechanical axis deviation (MAD) was quantified by measurement of the distance between the mechanical axis of the lower extremity and the intercondylar eminence of the tibia using a standing teleoroentgenogram.^15^ The amount of lateral shift of the axis from the neutral line was allocated positive value. We also measured mechanical lateral distal tibial angle (mLDTA) to evaluate ankle valgus deformity at the final follow-up according to Paley method.^15^

The pattern of callus shape was classified into 5 categories (fusiform, cylindrical, concave, lateral, and central) based on the callus diameter according to Li et al's classification.^16^ The maturity of callus was evaluated based on the radiolucent bone absorption within the hard callus. The mature callus was defined as the appearance of patchy radiolucencies as well as the formation of at least 1 continuous cortex on both the anteroposterior and lateral radiographs.

Successful lengthening was defined as the HI <50 days/cm without the associated fracture, and unsuccessful lengthening was defined as either the HI ≥50 days/cm or the presence of the associated fracture.

### Statistical Analysis

Clinical and demographic variables and radiological measurements were as follows: the qualitative/categorical variables are gender, affected side, the kind of bone distracted, Achterman–Kalamchi classification, callus shape and maturity, and occurrence of regenerating bone fracture; the quantitative variables are age, LLD, length gained, the number of the concomitant abnormalities, degree of tibial medial bowing, and MAD. Data were collected and analyzed using the IBM statistics SPSS version 22 (Chicago, IL). The Mann–Whitney *U* test was used to examine significant differences with respect to age at surgery and the number of the congenital anomalies between successful and unsuccessful lengthening. The association between the incidence of regenerate fracture versus lengthening ratio and the incidence of regenerate fracture versus callus maturation were examined with Fisher exact probability test. Differences were considered statistically significant when *P* <0.05.

## RESULTS

Demographic information and patient characteristics are summarized in Table 1. Eight patients (5 males and 3 females) with no bilateral involvement were included in this study. The right leg was involved in 7 and the left in 1. A total of 13 bone lengthenings were performed including 1 femoral single lengthening, 2 tibial single lengthenings, 3 concomitant lengthenings of the femur and tibia, and 2 staged lengthenings of the femur and tibia. As for the staged lengthening, the initial lengthening was conducted on the femur in one and on the tibia in one. The mean ages at surgery and the final follow-up are 13.2 years (range 5.3–21.9) and 17.3 years (range 12.1–27.4), respectively. The average duration of follow-up after the initial lengthening was 55 months (range 15–94). According to the Achterman–Kalamchi classification, the patients were classified into type Ia in 5, Ib in 2, and II in 1. Preceding surgeries were performed in 2 patients for resistant equinovarus deformity of the foot. One is posterior release at the age of 7 months and another is posterior release and partial excision of the distal fibular vestige at the age of 13 months. Patient 5 had undergone femoral varus derotational osteotomy and rotational acetabular osteotomy in the left hip due to residual acetabular dysplasia associated with developmental dislocation of the hip at 6 years and 16 years of age, respectively.

**TABLE 1:**
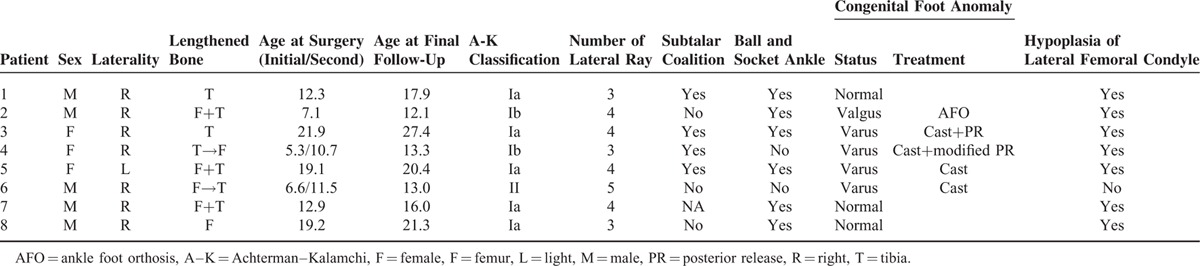
Demographics Information and Patient Characteristics of the Study Population

Clinical information on bone lengthening is summarized in Table 2. The mean amount of LLD at surgery and the final follow-up are 48 mm (range 20–70) and 5 mm (range 2–11), respectively. The average length gained and HI are 48 mm (range 25–80) and 72.9 days/cm (range 24.5–170.5), respectively. The shape of callus was fusiform in 2, cylindrical in 9, concave in 1, and lateral in 1. There were 4 regenerate bone fractures, 3 of them occurred before removal of the bone screws, and 1 occurred after removal of the bone screws. All fractured bone had cylindrical callus. They were treated by cast immobilization or reattachment of the fixator, but healed with deformity in 2. In patient 2, considerable anterolateral bowing progressed at the fracture site following reattachment of the fixator due to fatigue loosening of the bone screws. He sustained the second femoral fracture at the distal host-regenerate junction by falling 2 years after the first fracture. He underwent corrective osteotomy and intramedullary Kirschner wire fixation. In patient 4, anterior bowing of the tibia progressed during cast immobilization, and she needed corrective osteotomy at 9 years of age.

**TABLE 2:**
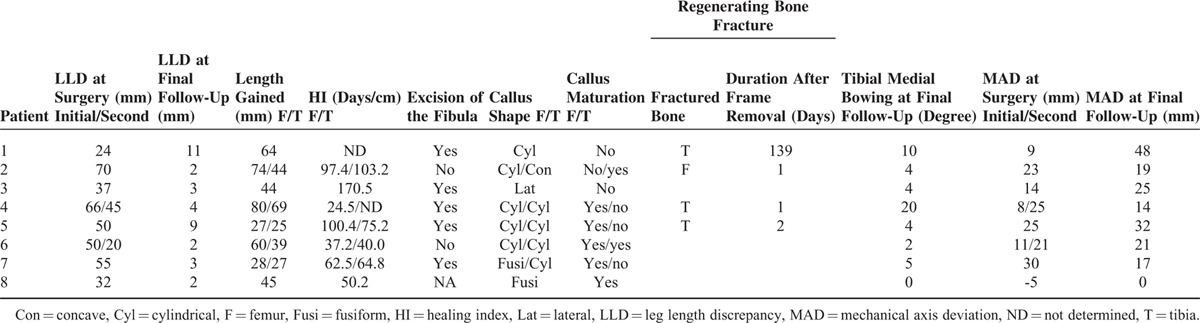
Clinical Information on Bone Lengthening

There was a significant difference in age at surgery (*P* = 0.042) between successful and unsuccessful lengthening, but not in the number of the congenital anomalies (*P* = 0.078). The incidence of regenerate fractures was significantly correlated with the callus maturity before the frame removal (*P* = 0.021; Tables 2 and 3). Patients with the lengthening ratio ≥20% were more susceptible to the regenerate fractures than those with the lengthening ratio <20%, although the difference was of borderline significance (*P* = 0.052; Table 4). LLD was successfully corrected within 11 mm, whereas mechanical axis deviated laterally in all cases at the final follow-up (Table 2). Residual valgus deformity of the tibia was observed in 6 patients. Valgus deformity of the ankle greater than 80 degrees of mLDTA was shown in 6 patients at the final follow-up.

**TABLE 3:**
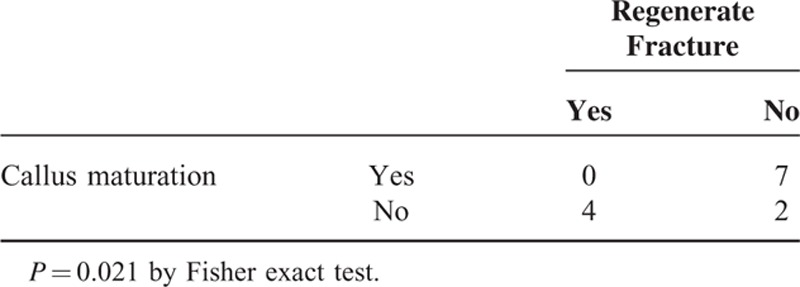
Relationship Between Callus Maturation and Regenerate Fracture

**TABLE 4:**
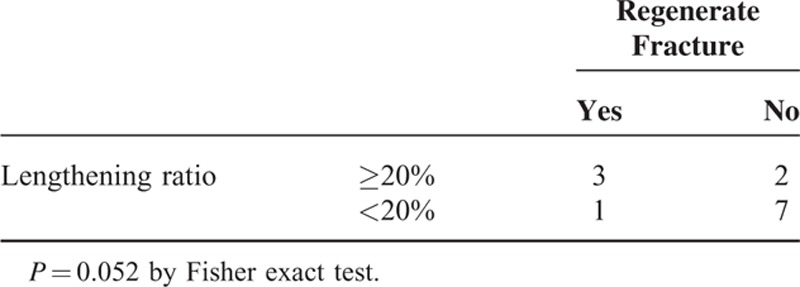
Relationship Between Lengthening Ratio and Regenerate Fracture

## DISCUSSION

The mean HI in the present study was larger than that in most of the previous reports, which showed around 50 days/cm.^8–11^ However, we obtained satisfactory correction of LLD in all cases, although most of the limbs in the previous reports remained clinically significant LLD at the latest follow-up.^8–11,17,18^ These results indicated that extended treatment period would be needed to secure acceptable correction of LLD probably because of underlying poor capacity of bone regeneration. Since the age at surgery correlated with successful lengthening, earlier bone lengthening may be favorable for patients with LLD in fibular hemimelia.

Higher prevalence of congenital abnormalities is deemed to be associated with poor clinical and radiological outcomes in fibular hemimelia.^3,19–22^ However, Rodriguez-Ramirez et al^6^ recently reported that the number of congenital abnormalities is not a predictor of the final LLD in this disorder. In an agreement with their study, associated congenital abnormalities did not correlate to the outcome of the procedure. Successful correction of LLD could be achieved by bone lengthening even in cases with higher prevalence of the limb anomalies.

Some authors have emphasized that a narrow-shaped callus had a higher risk of regenerate fractures following bone lengthening.^23,24^ Our fracture cases were, however, not associated with the callus shape but with the callus maturity. Advanced maturation of the callus seemed to be important to avoid the regenerate fractures in this specific disorder. A previous study noted that the number of the fractures in fibular hemimelia increased when the lengthened ratio exceeded 15% of the original length of the bone.^25^ Similar relationship between the lengthened ratio and the regenerate fracture was found in our study. The optimal amount of lengthening and the timing of the fixator removal should be determined based on the lengthened ratio and the remodeling status of the regenerate.

Fibular hemimelia tends to exhibit genu valgus deformity of the affected limb due to hypoplasia of the lateral femoral condyle and anteromedial bowing of the tibia during growth.^2^ The lengthened tibia is more prone to bend medially presumably because of soft tissue tightness of the lateral component of the lower leg. The fibrocartilaginous anlage of the fibula is believed to tether from the proximal part of the tibia to the posterolateral calcaneus, which causes the anteromedial bowing of the tibia during lengthening.^26^ Severe valgus deformity of the ankle may be correlated with persistent anteromedial bowing of the tibia after lengthening.^8^ A slight overcorrection of the genu valgum is thus preferred during the growth period, as has been proposed by Zarzycki et al,^11^ to minimize the possibility of axial deviation. A recent report has documented favorable correction of genu valgum and increased lower limb function by 8-plate hemiepiphysiodesis around the knee in this disorder.^27^ Correction of the mechanical axis also contributes to the reduction of lengthening to resolve LLD. More careful attention is required for the attachment of external fixator and the positioning of osteotomy site in tibial lengthening for fibular hemimelia, because a tibial lengthening with a monolateral fixator commonly causes valgus malalignment during and after the lengthening when the osteotomy is done more distally and the fixator is not placed parallel to the mechanical axis.^28^ To maintain and correct the mechanical axis during limb lengthening, the use of a circular fixator has been advocated especially for the cases with large fibular defect.^9,11,29^

Our study has some limitations. First, LLD in this study represented a length from the femoral head to the ankle joint and did not include the thickness of the foot. Because the discrepancy of the foot thickness is commonly involved in fibular hemimelia, the measurement of LLD may be preferable by an indirect method using blocks in standing posture.^13^ Second, the sample size was small because of its rarity. We could not draw definitive conclusions regarding the optimal timing of bone lengthening. Third, due to the retrospective nature of the data, pain and functional outcomes before and after the bone lengthening could not be examined.

In conclusion, our study demonstrated that limb lengthening enables satisfactory resolution of clinically significant LLD in fibular hemimelia, but inevitably involves the major complications, including delayed maturation of the lengthened callus, regenerating bone fractures after the fixator removal, and valgus malalignment of the affected limb. We practitioners should pay attention to the status of callus remodeling during limb lengthening and the mechanical axis deviation throughout the treatment period in fibular hemimelia.
